# How do we work with medical teams to provide optimal treatment for clients in the acute medical environment?

**DOI:** 10.1186/2050-2974-2-S1-O25

**Published:** 2014-11-24

**Authors:** Lisa Stokes, Michael Bruce

**Affiliations:** 1 Royal Melbourne Hospital, Melbourne, Australia

## 

Historically, there have always been medically unwell clients with eating disorders whose pathway of care involves presentations and admissions to emergency departments, intensive care units and/or medical/surgical units. At the Royal Melbourne Hospital there has been a noted increase in not only the number of clients requiring admissions to medial units, but also in the severity in medical acuity of these clients.

Lack of clinical expertise in the acute health sector can result in delayed or sub optimal treatment, increased length of stay and poorer client outcomes.

This paper will outline a new initiative proposed at the Royal Melbourne Hospital involving the collaboration of Consultation Liaison Psychiatry, Specialist Services Eating Disorders Program and an Acute Medical Unit. The aim of this project is to support staff in the acute health sector, particularly nurses, in their understanding of the psychopathology of eating disorders, to improve clinical skills and provide timely and appropriate interventions to clients and their families.

The core of this project is the development of an E-learning package, specific to the detection, assessment management and ongoing care of clients with eating disorders in the acute health sector.

This abstract was presented in the **Service Initiatives** stream of the 2014 ANZAED Conference.

